# Human genetic determinants of COVID-19 in Brazil: challenges and future plans

**DOI:** 10.1590/1678-4685-GMB-2023-0128

**Published:** 2024-01-15

**Authors:** Bibiana S. de Oliveira Fam, Marilea Furtado Feira, Nathan Araujo Cadore, Renan Sbruzzi, Tábita Hünemeier, Laurent Abel, Qian Zhang, Jean-Laurent Casanova, Fernanda Sales Luiz Vianna

**Affiliations:** 1 Hospital de Clínicas de Porto Alegre, Centro de Pesquisa Experimental, Laboratório de Medicina Genômica, Porto Alegre, RS, Brazil.; 2 Universidade Federal do Rio Grande do Sul, Instituto de Biociências, Departamento de Genética, Laboratório de Imunogenética, Programa de Pós-Graduação em Genética e Biologia Molecular, Porto Alegre, RS, Brazil.; 3 Instituto Nacional de Genética Médica Populacional (INaGeMP), Porto Alegre, RS, Brazil.; 4 Universidade de São Paulo, Instituto de Biociências, Departamento de Genética e Biologia Evolutiva, São Paulo, SP, Brazil.; 5 Institut de Biologia Evolutiva (Consejo Superior de Investigaciones Científicas/Universitat Pompeu Fabra), Barcelona, Spain.; 6 The Rockefeller University, Rockefeller Branch, St. Giles Laboratory of Human Genetics of Infectious Diseases, New York, NY, USA.; 7 Howard Hughes Medical Institute, New York, NY, USA.; 8 Laboratory of Human Genetics of Infectious Diseases, Imagine Institute, Paris, France.; 9 University Paris Cité, Imagine Institute, Paris, France.; 10 Necker Hospital for Sick Children, Department of Pediatrics, Paris, France.

**Keywords:** COVID-19, Brazilian population, inborn errors of immunity, autoantibodies type I IFN, host genomic

## Abstract

COVID-19 pandemic represented a worldwide major challenge in different areas, and efforts undertaken by the scientific community led to the understanding of some of the genetic determinants that influence the different COVID-19 outcomes. In this paper, we review the studies about the role of human genetics in COVID-19 severity and how Brazilian studies also contributed to those findings. Rare variants in genes related to Inborn Errors of Immunity (IEI) in the type I interferons pathway, and its phenocopies, have been described as being causative of severe outcomes. IEI and its phenocopies are present in Brazil, not only in COVID-19 patients, but also in autoimmune conditions and severe reactions to yellow fever vaccine. In addition, studies focusing on common variants and GWAS studies encompassing worldwide patients have found several loci associated with COVID-19 severity. A GWAS study including only Brazilian COVID-19 patients identified a new locus 1q32.1 associated with COVID-19 severity. Thus, more comprehensive studies considering the Brazilian genomic diversity should be performed, since they can help to reveal not only what are the genetic determinants that contribute to the different outcomes for COVID-19 in the Brazilian population, but in the understanding of human genetics in different health conditions.

## Introduction

COVID-19, one of the diseases triggered by infection by SARS-CoV-2, presents a high variability of clinical manifestations and outcomes. Before the vaccination became widely available, around 10% of the patients progressed to hypoxemic pneumonia, 3% to severe acute respiratory distress syndrome (ARDS), and 1% to death (Zhang et al., 2022a). After three years of the pandemic and even with fast vaccine development, more than 760 million cases and 6.8 million deaths worldwide have been recorded (WHO, 2023). Brazil is one of the most affected countries by COVID-19, being the fifth in number of cases (37.4 million) and second in number of deaths (700,000) (Coronavírus Brasil, 2023). The main epidemiological risk factors for severity and death in the Brazilian population are in accordance with worldwide studies and include primarily age over 60 years old, and to a much lower extent, lower socioeconomic status, male gender, and presence of comorbidities (Zhang *et al.*, 2020a; Sansone et al., 2022). In addition to these, Sansone *et al.* (2022) analyzed 585,655 Brazilian hospitalized patients and identified that Native Americans, Afro-descendants, and multiracial background individuals were slightly more likely to die from COVID-19, when compared with white Brazilians, with odds 1.91 (95% CI = 1.70-2.15), 1.43 times (95% CI = 1.39-1.48), and 1.36 (95% CI = 1.34-1.38), respectively. Although these are very low risks, the association among ethnic groups in the Brazilian population was also observed in young patients. Soares et al. (2022) analyzed children and adolescents hospitalized due to COVID-19 and identified higher mortality in Native Americans (3.2-fold), and in patients from as North (3.9-fold) and Northeast (5.9-fold) of Brazil. It is fundamental to highlight that the access to health care and socioeconomic status play a major role in these findings, since with adequate treatment, mortality from pneumonia cases decreases drastically (Zar et al., 2013). Nevertheless, even with adequate health care, several other aspects have also been attributed to the remarkable variability observed in the response to COVID-19, especially variability of the human genome. Numerous studies worldwide have been performed regarding the role of host genetics in the COVID-19 clinical response. Some of them have provided compelling evidence about the importance of genetic variants in the disease, which has helped to better understand the mechanisms of the disease. However, the impact and prevalence of rare and common variants in genes important in the COVID-19 response can change due to the genetic variability of the population studied, implying potentially different risks for each region of the globe (Kehdy et al., 2015; Secolin et al., 2019; Zhang *et al.*, 2022a; Cobat et al., 2023). 

Brazil is the fifth-largest country by area and the seventh most populous country, with almost 216 million residents (IBGE, 2023). The Brazilian population has ethnic and cultural diversity and a unique genetic profile, formed by Native Americans, Europeans, and Africans, resulting in a heterogeneous and admixture population (Kehdy et al., 2015; Secolin et al., 2019). In addition, the colonization associated with geographic and social processes also favored the formation of isolated populations groups with different genetic characteristics due to geographic isolation, endogamy, size, and effectiveness of the reduced population, being some of them have a high prevalence of genetic disorders (Cardoso et al., 2019). This complex genetic pattern encompasses both common and rare variants, which can have an impact on several health conditions, including COVID-19 and other infectious diseases (Cobat et al., 2023). Indeed, it has already been observed that isolated populations and private genetic variants of some populations present different impacts depending on the region and infections analyzed (Bastard et al., 2022a; Duncan et al., 2022; Couto-Silva et al., 2023). In this paper, we will review studies focused on human genome-wide analyses and their contribution to COVID-19 outcome understanding, as well as the Brazilian studies on those discoveries. From these findings, we discuss the challenges and opportunities from a huge, populous, admixture, and lower-middle income country for genetic studies on infectious diseases.

### Host genetics can explain, at least in part, the inter-individual variability to infections

The outcomes after exposure to a pathogen in humans can range from resistance to infection to death, and throughout decades, it has been described several hundreds of examples of this spectrum related to human host genetics (Casanova and Abel, 2021a). Well-known examples of partial resistance to infectious disease include sickle cell trait and resistance to malaria (Allison, 1954), and the common deletion of 32 nucleotides within the *CCR5* gene (*CCR5*-Delta32 allele; rs333), which confers complete resistance to the Human Immunodeficiency Virus 1 (HIV-1) (Dean et al., 1996). Although there are other examples of genetic resistance to infections, the number of studies regarding the role of genetic variants on the severity of infections is also striking. Indeed, it is essential to mention that life-threatening disease is a rare outcome for most infections (Casanova and Abel, 2021b). In such rare severe cases, the risk caused by germline mutations has been consistently demonstrated. The first mutated gene identified associated with the phenotype of severe infectious disease was the *ADA* gene in 1985 in patients with severe combined immunodeficiency (characterized by multiple and severe infections) (Bonthron et al., 1985). Since then, it was possible to identify the molecular basis for several types of infectious diseases, including rare cases of infectious diseases with mendelian inheritance, as well as rare monogenic defects underlying some common infectious diseases (reviewed by Casanova and Abel, 2022). All those findings have allowed the understanding not only of several genetic determinants involved in infectious diseases but also of a group of diseases called Inborn Errors of Immunity (IEIs), previously known as primary immunodeficiencies. These studies had a central role in understanding the genetic determinants of COVID-19.

### Inborn errors of immunity can underlie severe COVID-19 pneumonia

IEIs are a heterogeneous group of more than 430 rare monogenic diseases that predispose individuals to severe infections, autoimmunity, and autoinflammation (Tangye et al., 2022). One specific type of IEI crucial to uncover genetic determinants of COVID-19 are inborn errors of type I interferon (IFN) immunity (Casanova and Anderson, 2023). These conditions are characterized by pathogenic variants in genes governing type I IFN immunity, an essential response pathway to viral infections. The first approach used in COVID-19 was focused on type I IFN genes already described to cause severe pneumonia (Zhang et al., 2022a). A study from the COVID Human Genetic Effort (https://www.covidhge.com) analyzed a sample of 659 individuals with life-threatening COVID-19 and observed that at least 23 (3.5%) of the patients had loss-of-function mutations in 8 out of 13 tested *loci* (*IRF3, IRF7, IFNAR1, IFNAR2, TLR3, TICAM1, TBK1,* and *UNC93B1*) (Zhang *et al.*, 2020b). Other investigations showed inborn errors of type I IFN, especially in young men with severe COVID-19. A case series of pairs of brothers otherwise healthy, which were admitted to ICU due to COVID-19, showed rare putative loss-of-function variants in *TLR7* in four cases (van der Made *et al.*, 2020). In another cohort of 1,202 males with critical pneumonia, 17 patients (1.4%) had biochemically proven deleterious *TLR7* mutations (Asano et al., 2021). Further studies confirmed IEI as a cause of severe COVID-19 in different populations, including children (Campbell et al., 2022; Zhang *et al.*, 2022a). Those studies provided evidence that IEI with rare germline mutations in different type I IFN genes can cause severe outcomes of COVID-19. These findings were recently replicated on a larger scale (Matuozzo et al., 2023). Moreover, they have demonstrated the same biological mechanism related to other infections with similar phenotypes.

### Autoimmune phenocopies of inborn errors of type I IFN immunity pathway also cause severe COVID-19

Following the biological mechanisms related to the type I IFN pathway, the role of autoantibodies targeting type I IFN, an autoimmune phenocopy of IEI, was also hypothesized and evaluated in COVID-19 patients. The first study to explore type I IFN autoantibodies was published by Bastard et al. (2020), including 987 patients with life-threatening COVID-19 pneumonia, 663 mild or asymptomatic cases, and 1,227 healthy controls. IgG autoantibodies targeting type I IFNs were detected in 13.7% (135) of individuals with life-threatening COVID-19. It was also observed that in 52 of these patients, the detected autoantibodies were neutralizing both IFN-α2 and IFN-ω, and only IFN-α2 or IFN-ω in 36 and 13 patients, respectively. No type I IFN autoantibodies were detected in the 663 mild or asymptomatic COVID-19 cases, and they were found in only 4 of 1,227 healthy individuals (Bastard *et al*., 2020). These auto-Abs are present in the general population (Bastard *et al.*, 2021). Another study with 1,261 unvaccinated patients deceased due to COVID-19 and 34,159 individuals from the general population analyzed the presence and titers of type 1 IFN neutralizing autoantibodies, determining relative risk of death (RRD) and infection fatality rate (IFR). It was observed that the RRD produced by any combination of autoantibodies was higher in individuals in patients under 70 years (RRD: 17; 95% CI: 11.7 to 24.7), and IFR increase with age (40.5% for >80 yo; CI95% 27.82 to 61.20) for autoantibodies neutralizing both IFN-α2 and IFN-ω (Manry et al., 2022). These auto-Abs have been found in at least 20 other independent studies (reviewed in Zhang et al., 2022a; Casanova and Anderson, 2023). The presence of neutralizing autoantibodies against type I IFN has been detected in around 10-20% of critical cases of COVID-19 pneumonia (reviewed by Puel et al., 2022).They were also found to underlie 24% of vaccine-breakthrough COVID-19 pneumonia (Bastard *et al.*, 2022b), 25% of cases of critical MERS pneumonia (Alotaibi et al., 2023) and 5% of cases of critical influenza (Zhang *et al.*, 2022b).

### The phenotype of rare inborn errors of type I IFN immunity in Brazil is revealed by severe reactions to Yellow Fever Vaccine (YFV)

Although comprehensive studies regarding the prevalence of inborn errors of type I IFN in Brazilian COVID-19 patients had not been localized, previous studies on YFV severe reactions have shown interesting findings, which can also have an impact to COVID-19. YFV reactions can, in rare cases, cause life-threatening reactions (Lindsey et al., 2016). In 2019, Hernandez et al. (2019) evaluated a Brazilian 14 years-old girl considered healthy until she was 12 years old, when she received the YFV and presented a severe reaction seven days after the vaccination. Whole exome sequencing (WES) identified a compound heterozygous loss-of-function mutation in *IFNAR1* gene as the cause of the YFV reaction. Later, Bastard et al. (2021) identified a homozygous variant in the *IFNAR2* gene associated with complete IFNAR2 deficiency as the cause of reaction to YFV in a Brazilian woman who suffered from a severe YFV reaction at the age of 13. She had no previous history of severe viral infections, but interestingly, her sister died from severe reaction to YFV at the age of 19. Although germline mutations in the patient’s sister were not evaluated by the study, is it possible that the YFV reaction could have been caused by the same *IFNAR2* mutation. Likewise, we can hypothesize that these rare cases also could be at high risk for severe COVID-19, as well other severe viral illnesses, through the pathogenic mechanisms of complete IFNAR deficiency. In addition, other Brazilian individuals may be carrier pathogenic variants in inborn errors of type I IFN genes in the population, however, they may not have been identified due to the limited studies performed until now.

### Autoantibodies against type I IFN are present in Brazilian patients with YFV reactions, autoimmune diseases, and COVID-19

Following the hypothesis of the mechanism observed for severe cases of COVID-19, Bastard et al. (2021) also analyzed if autoantibodies against type I IFNs might be causal in life-threatening diseases related to YFV that did not present germline mutation in IEI genes. They identified that among eight patients with YFV severe reactions, three had high titers of circulating autoantibodies against type I IFNs, and one of them was a Brazilian. Interestingly, this 50 years-old woman was also diagnosed with lupus erythematosus systemic (SLE) during her hospital stay to treat YFV reaction. SLE patients have been reported to produce autoantibodies against type I IFN (Puel et al., 2022), and they also have been considered at risk of adverse reactions to YFV (Seligman, 2014). Another study with Brazilian patients reported by Schidlowski et al. (2022), described two brothers, 13 and 7 years, who presented life-threatening COVID-19 pneumonia, and after a deeper investigation were diagnosed with polyendocrinopathy syndrome type I (APS-1), an also known condition to produce autoantibodies against type I IFNs. Prior to SARS-CoV-2 infection, they had not presented the classical phenotype of this IEI, but the disease was considered, and confirmed, after WES performed due to clinical phenotype presented by them during the COVID-19 (Schidlowski *et al*., 2022). Moreover, in a study that evaluated 121 Brazilian IEI patients who had COVID-19, it was observed one of them had Good syndrome and died of COVID-19 (Goudouris et al., 2021). Although the authors did not mention the presence of neutralizing autoantibodies to type I IFN in that patient, they are common in Good syndrome and likely represent a risk factor for severe COVID-19 (Delmonte et al., 2022). Taken altogether, these findings provide evidence that Brazilians with autoimmune diseases, reactions to YFV or even previously healthy would be at risk to severe COVID-19 due to presence of autoantibodies against type I IFN. We can also hypothesize that this could have been the cause, in some proportion, of the hospitalizations and deaths of COVID-19 patients in this country.

### Common genetic variants also may contribute to COVID-19 severity

Common genetic variants have also been evaluated regarding the predisposition of SARS-CoV-2 infection and COVID-19 outcomes. Common variants (minor allele frequency >1% of the population) throughout the genome can contribute to the pathogenesis of complex diseases (Kwok et al., 2021). Advances in technology have greatly benefited common genetic variant studies, and genome-wide association studies (GWAS) have been widely used to identify host genetic variants in the context of infectious diseases (reviewed by Kwok *et al*., 2021). Thus, worldwide efforts have been taken during the pandemic of COVID-19 to conduct genomic investigations on common variants, resulting in the identification of several *loci* associated with differential outcomes of COVID-19 (Cobat et al., 2023). The first and most significant *locus* described in GWAS associated with COVID-19 critical is 3p21.31, which encompasses six genes (*SLC6A20*, *LZTFL1*, *CCR9*, *FYCO1*, *CXCR6*, and *XCR1*) (The Severe COVID-19 GWAS Group, 2020). This association was also identified in further GWAS studies (Pairo-Castineira et al., 2021; Shelton et al., 2021; COVID-19 Host Genetics Initiative, 2021; Horowitz et al., 2022; Kousathanas et al., 2022; Degenhardt et al., 2022). Another important locus reported by GWAS studies is 12q24.13, spanning three genes (*OAS1*, *OAS2*, and *OAS3*) which encode antiviral restriction enzyme activators. In addition, a 19p13.2 locus, where the *TYK2* gene is located, was also associated with patients with critical COVID-19 (Pairo-Castineira *et al.*, 2021; COVID-19 Host Genetics Initiative, 2021; Horowitz *et al.*, 2022; Kousathanas *et al.*, 2022; Degenhardt *et al.*, 2022). Moreover, a GWAS including European individuals and multi-ancestry meta-analysis performed by Kousathanas *et al.* (2022) showed an association with human leukocyte antigen (HLA) locus, upstream *HLA*-*DQA1* and *HLA*-*DRB1* genes. 

Some of the loci detected by GWAS point to a role for genes with relevant biological functions in the context of SARS-CoV-2 infection. However, fine mapping the causal variants has been challenging for many reasons. Common variants present a very modest effect on the outcomes, with the odds ratio generally lower than 2 (Fricke-Galindo and Falfán-Valencia, 2021). Besides, accuracy in defining the phenotype of interest is essential for the identification of associations and replication of the results in other cohorts (Park et al., 2010). Moreover, it is fundamental to mention that most of the studies show a high number of European-ancestry samples, which can bias the identification of association gene-trait in other populations, since GWAS findings are based on Linkage Disequilibrium (LD) patterns (Hayes, 2013). One example is the 3p21.31 region, which displays LD structure and patterns of association that suggest untagged genetic variation could drive the association signal (COVID-19 Host Genetics Initiative, 2021). Non-European studies or studies that were stratified by population-specific have identified loci previously unreported (Pairo-Castineira et al., 2021; Secolin et al., 2021; Pereira et al., 2022). Thus, the lack of representativeness of multiple ancestries in GWAS studies can be considered a limiting factor in these studies and must be considered when analyzing associations between markers and putative causative variants. Together these findings indicate that frequent variations in several regions and genes spread across the genome can play a role in COVID-19, and shed light on the severity mechanisms of the disease-related and also independent of type I IFN. 

### Some common genetic variants associated with COVID-19 were found only in Brazil

Only one GWAS study was performed focused specifically on the Brazilian population. Pereira et al. (2022) compared 3,533 hospitalized with 1,700 non-hospitalized COVID-19 patients and identified a new locus 1q32.1 associated with COVID-19 severity, which spanned the genes *CNTN2*, *TMEM81*, *RBBP5*, *DSTYK*, and *TMCC2*. Although the signal had been associated only in Brazilians with high European ancestry, it was not described by previous studies, suggesting this region might harbor a population specific risk allele or haplotype. The group also investigated the phenotypes previously described as associated with the tag-SNP for this locus (rs11240388), and these were mainly related to hematological traits, including immune cell count and morphology. Interestingly, Upadhyai et al. (2022) found an association of common coding variants in two genes of this region (*CNTN2* and *TMCC2*) also evaluating individuals of European ancestry. Finally, one WES-based Brazilian study focused on identifying associations related to HLA alleles with COVID-19. Castelli et al. (2021) identified the association between the *HLA-DRB1* alleles encoding K at residue 71 (DRB1*03:01, DRB1*04:01, and others) and the HLA-DOB*01:02 allele with symptomatic infections. Important to note that *HLA-DRB1* genetic variants were already found in GWAS analysis, as shown by Kousathanas et al. (2022). Therefore, the variability of *HLA* alleles may contribute to the progression of COVID-19. However, the genetic diversity of *HLA* genes is still not well understood in the context of COVID-19, in particular in the Brazilian population which has a very diverse ethnic origin. These results demonstrate the need for the inclusion of genetic diversity on GWAS of COVID-19 and *HLA* investigations. 

### Challenges and perspectives of Brazilian science facing the COVID-19 pandemic

The numbers of the COVID-19 in Brazil have been impressive in cases and deaths, making the country the epicenter of the COVID-19 pandemic in Latin America. Facing the pandemic has shown the country’s broad scientific capacity, while at the same time revealing a series of needs that still need to be addressed in order to develop the country’s full potential. Since the beginning of the pandemic, traditional Brazilian researchers and institutions such as Butantan Institute, Fiocruz, Fundação Oswaldo Cruz, and Universities have been intensely dedicated to combating SARS-CoV-2 and understanding COVID-19. This collaborative effort by the Brazilian scientific community resulted in different studies, contributing to advances in genomic and epidemiological surveillance of the virus, vaccine development, diagnostic tests, and treatments for the disease (Castro et al., 2021). In investigations of the role of human genetic variants on COVID-19, Brazilian researchers have also been part of the main international consortia, such as the COVID Human Genetic Effort (https://www.covidhge.com/) and COVID-19 Host Genetics Initiative (https://www.covid19hg.org), which have revealed instrumental evidence to understand the disease’s mechanisms. Brazilian centers in the Southeast and South of Brazil have been part of the collaborative networks and contributed with samples from Brazilian patients in several publications related both with to IEI and its autoimmune phenocopies (Bastard et al., 2020; Goudouris et al., 2021; de Castro et al., 2022; Schidlowski et al., 2022; Manry et al., 2022; Zhang et al., 2022b; Matuozzo et al., 2023) and also common variants (Castelli et al., 2021; Castelli *et al.*, 2022; Pereira et al., 2022; Secolin et al., 2021). However, Brazilian initiatives using genome-wide approaches for other Brazilians regions have not also been numerous. Thus, considering the impact of the COVID-19 pandemic, the size of the population and the Brazilian genetic variability, determinants important in the COVID-19 response may be being missed due to a lack of genetic studies in the country, which could be an essential piece of the COVID-19 puzzle.

One known case that demonstrates the importance of nationwide evaluation of genetic diversity is exemplified by studies with the *CCR5*-Delta32 allele. The South and Southeast Brazilian regions, which have a greater component of European ancestry, present a higher frequency of the allele (reaching ~9%), while the North, Northeast, and Southeast regions present lower frequencies (~2%) (Kulmann-Leal et al., 2021). In Brazilian isolated populations, this allele is considered rare, being absent in certain isolated Native American populations, or with a low frequency being introgressed through miscegenation with Europeans (Hünemeier et al., 2007). As well as the *CCR5*-Delta32 allele, other examples enhance the need for a comprehensive exploration of the genetic architecture of the Brazilian (Nunes et al., 2021). However, the challenge related to understanding and generating genomic data of the Brazilian population is also hampered by the high socio-economic inequality and a low-financial resource setting, both in research and medical assistance scenarios. The high costs and low availability of the technologies using next-generation sequencing approaches in diagnosis and in clinical practice investigations not only prevent identifying known or novel genes related to phenotypes in infectious diseases but also hinder the offering of better diagnostic, and therapeutic options in Brazil. Thus, large-scale investment in genomic sequencing projects and national genomic database implementation that allow the identification of rare and common variants, as well as the comparison among Brazilian regions and other populations must be strongly encouraged and prioritized. Some Brazilian initiatives, such as “DNA do Brasil” (Patrinos et al., 2020) and The Brazilian Initiative on Precision Medicine (BIPMed) (Rocha et al., 2020), and ABraOM: Brazilian genomic variants (https://abraom.ib.usp.br/) are already committed to addressing these issues. This genomic information will be useful in health care and also in research, helping in the identification of genes or variants underlying common and rare phenotypes, as well as driving precision medicine approaches population-specific.

## Concluding remarks

Throughout the pandemic, we observed an unprecedented worldwide effort to understand different aspects of COVID-19 severity. Studies evaluating rare genetic variants in genes governing type I IFN or its autoimmune phenocopies have found them to cause around 15% of cases of severe COVID-19 pneumonia. No studies with a similar approach specifically addressed the Brazilian population with COVID-19. Nevertheless, from a few Brazilian cases already described in the literature with autoimmune diseases and YFV reactions, it is possible to speculate that inborn errors of type I IFN immunity and its phenocopies could be present in COVID-19 Brazilian patients, as well as in other patients with severe viral diseases and autoimmune conditions, or even in healthy people. This hypothesis brings up the necessity of a comprehensive evaluation not only of COVID-19 but also of patients with other conditions, which can be at risk for severe viral diseases and reactions to live-attenuated vaccines. A deeper investigation of Brazilian patients may provide a more realistic estimation of those conditions in the country, besides helping to plan strategies to screen and properly manage patients. Additionally, research focused on families and Brazilian isolated populations can reveal if private mutations on IEI genes are present in Brazil. In this sense, although the common genetic factors underlying the severity of COVID-19 have been extensively studied worldwide through multiple GWAS, the example of the single GWAS conducted specifically on the Brazilian population demonstrates the importance of considering population-specific genetic variations when searching for genetic determinants of infectious diseases. Thus, combining all those strategies with other efforts focused on genome-wide common variants may be useful for expanding the knowledge about genetic modifiers and other biological pathways determinants of disease. Finally, the identification of variants associated with a severe outcome in infections can drive actions and specific prevention guidelines for risk groups. Knowledge about the genetic basis of infectious diseases may allow the development of more effective or precision medicine therapies for individuals affected.
